# Comparison of Autorefraction and Photorefraction with and without Cycloplegia Using 1% Tropicamide in Preschool Children

**DOI:** 10.1155/2019/1487013

**Published:** 2019-05-02

**Authors:** Ertuğrul Tan Yassa, Cihan Ünlü

**Affiliations:** ^1^MD, FEBO, Asya Eye Medical Centre, Ophthalmology Department, Istanbul 34310, Turkey; ^2^MD, Asya Eye Medical Centre, Ophthalmology Department, Istanbul 34310, Turkey

## Abstract

**Purpose:**

We aimed to investigate whether the accuracy of the Plusoptix A09 photorefractor in children with ametropia is enhanced by cycloplegia with 1% tropicamide.

**Methods:**

A total of 70 eyes (70 children) were retrospectively reviewed. Noncycloplegic photorefraction, cycloplegia with 1% tropicamide, cycloplegic photorefraction, and cycloplegic refraction with a tabletop autorefractometer were performed on all subjects in this order. Measurements were compared statistically.

**Results:**

The mean age was 45.9 ± 11.4 months. The mean spherical equivalent (0.61 ± 1.03 diopters (D); range, −2.38 to 3.63 D) and mean spherical power (1.16 ± 0.92 D; range, −1.25 to 3.75 D) values that were acquired from the photorefraction without cycloplegia showed statistically significant differences from those of the autorefraction with cycloplegia (mean spherical equivalent = 1.00 ± 1.27 D; range, −1.50 to 4.25 D, mean spherical power = 1.60 ± 1.14; range, −1.25 to 4.50 D). The mean difference for the spherical equivalent was −0.39 ± 0.93 D (*P*=0.021; 95% limits of agreement (LoA) = −2.22 D to 1.44 D) and for spherical power was −0.44 ± 1.02 D (*P*=0.016; LoA = −2.44 D to 1.56 D). Without cycloplegia, Plusoptix A09 showed myopic shift, while after cycloplegia, it showed hyperopic shift. Spherical equivalent (mean difference (MD) ± SD = 0.78 ± 1.00 D, *P* < 0.001; LoA = −1.17 D to 2.72 D) and spherical power (MD ± SD = 0.73 ± 1.04 D, *P* < 0.001; LoA = −1.31 D to 2.77 D) values were significantly different from those of autorefraction with cycloplegia. Cylindrical power values obtained by photorefraction both with and without cycloplegia were not statistically different from those of autorefraction with cycloplegia (*P* > 0.05).

**Conclusion:**

Cycloplegia with 1% tropicamide did not improve the accuracy of photorefraction using Plusoptix A09 in preschool children. The spherical equivalent and spherical power values obtained by photorefraction with cycloplegia were significantly higher from those obtained by autorefraction with cycloplegia.

## 1. Introduction

Precise and early assessment of eye refractive errors is crucial because undetected anisometropia and high hyperopia may lead to amblyopia and promote strabismus. While there is some controversy as to the age when amblyopia becomes irreversible, there is consensus that the effectiveness of amblyopic treatment is greatest when initiated before the age of five [1]. On the other hand, obtaining accurate refractive error measurements in very young children continues to be a challenging exercise. In skilled hands, cycloplegic refraction using retinoscopy is still the most accurate method for examining children, and it represents the most commonly used gold standard for assessment. However, user dependency, the need for advanced clinical ophthalmic training, and being subject to interobserver variability are some of the limitations of retinoscopy [2]. The accuracy of the tabletop autorefractometers with cycloplegia for the refractive examinations of children has been established by the previous studies [3, 4]. However, maintaining a suitable position of the child and achieving visual fixation on a target for a sufficient length of time are some of the problems related to the utilisation of an autorefractometer [3]. Photorefraction may be a more suitable option for ophthalmologists who lack experience with retinoscopy and/or for children for whom proper evaluation is prevented due to the aforementioned issues.

Detailed refraction and pupil and ocular alignment measures are provided by the Plusoptix A09 (Plusoptix GmbH, Nurnberg, Germany) photorefractor series. Nonetheless, several studies have questioned its validity and reliability, reporting that even in its latest models, hyperopic refractive error is underestimated by the Plusoptix [5–9].

Accuracy of the photorefraction with cycloplegia has been investigated in some studies, and there has been controversy regarding their outcomes. Initially, with the use of cycloplegia in young and adult populations, the accuracy of cylinder power and axis decrease and the detection of hyperopia are enhanced, as demonstrated by Schimitzek and Lagrèze [9] On the other hand, incorrect results are obtained after application of cyclopentolate in the photorefraction measurements for determining refractive errors in children, as reported by Yilmaz et al. [10] and Ozdemir et al. [11].

Various studies have recommended tropicamide as a useful cycloplegic agent [12, 13]. Nevertheless, as far as we know, 1% tropicamide (as the cycloplegic agent) was not used exclusively by any of these studies, and photorefraction both with and without cycloplegia has been compared in few studies. In this study, we aimed to investigate whether cycloplegia with 1% tropicamide increased the accuracy of the Plusoptix A09 photorefractor in preschool children with ametropia. We compared the results obtained from photorefraction with and without cycloplegia with that of autorefraction with cycloplegia.

## 2. Methods

### 2.1. Patients

The Medical Ethics Committee of the Bakirkoy Dr. Sadi Konuk Education and Research Hospital of the Ministry of Health University approved the current study, and the study is in accordance with the Declaration of Helsinki.

The medical records of paediatric patients attending the Asya Goz Medical Center between October 2015 and April 2018 who had undergone photorefraction without cycloplegia, photorefraction with cycloplegia, and autorefraction with cycloplegia were retrospectively reviewed. The study population included all patients with complete medical records containing these data that had undergone a complete ophthalmologic examination, including a cover test and a fundus examination (with direct ophthalmoscope or slit-lamp biomicroscope depending on patient's age and cooperation). Photorefraction without cycloplegia, cycloplegia with 1% tropicamide, photorefraction with cycloplegia, and autorefraction with cycloplegia were performed in this order. Patients with eccentric fixation, significant media opacities, retinal abnormalities, refractive errors exceeding a cylindrical range of −7.00 to +5.00 diopters (D), and a spherical range of −7.00 to +5.00 D were excluded.

### 2.2. Devices

The Nidek ARK-510A autorefractor (Nidek, Gamagori, Japan) and the Plusoptix A09 distance photorefractor (Plusoptix GmbH, Nurnberg, Germany) were used. The tabletop Nidek ARK-510A autorefractor can measure the value of a sphere from –30 to +25 D in increments of 0.01 D and from 0 to 12 D for the cylinder in increments of 0.01 D. It can also measure small pupils down to 2 mm in diameter. The Plusoptix A09 is an infrared video camera that utilises eccentric photorefraction and determines the refractive status either binocularly or monocularly. A trained nurse operated the Plusoptix A09 photorefractor, which was placed at a distance of one meter facing the patient in a darkroom. It has a computer screen that shows the findings and the picture of the child. A handheld camera portion is attached to the computer, which gives both a moving light and a smiling-face fixation target with warble sounds. The reflected infrared light from the retina is evaluated by the camera. It can perform spherical and cylindrical refraction measurements within the range of −7.00 D to +5.00 D in increments of 0.25 D. It can perform refractive measurements within a pupil size range of 3 mm and 8 mm.

### 2.3. Cycloplegia

The cycloplegia application using 1% tropicamide (Tropamid®, Bilim Ilac, Istanbul, Turkey) was done as follows: two drops of 1% tropicamide separated by five minutes, were placed in the eye, and the refraction was measured twenty minutes after the second drop.

### 2.4. Statistical Analysis

Gold standards were defined by autorefraction with cycloplegia. To compare refraction measurements, the following techniques, which were established in prior studies, were used [14–18]. To determine the difference in mean spherical refractive error (DS), the noncycloplegic photorefraction result minus the cycloplegic autorefractometer result and the cycloplegic photorefraction result minus the cycloplegic autorefractometer result were calculated. The difference in the spherical equivalent refractive error (DSE) was calculated as follows: DSE = (*S*_*t*_ + 0.5 × *C*_*t*_) − (*S*_*c*_ + 0.5 × *C*_*c*_), where the spherical and the cylindrical powers are represented by *S* and *C*, respectively, and the control technique (cycloplegic autorefractometer) for comparison and the instrument being tested (photorefraction) are represented by the subscripts ‘*c*' (comparison) and ‘*t*' (test), respectively. A minus overestimation of the tested instrument is illustrated by a negative value for DS and DSE. The difference between the cylindrical powers (DC) was calculated as follows: DC = *C*_*t*_ − *C*_*c*_. The weighted cylindrical axis difference (DA) was calculated as follows: DA = 2 × *C*_*c*_ × sin (*α*_*t*_ − *α*_*c*_). In this formula, the difference between the cylindrical axes (test and comparison, measured in degrees) is weighted with the cylindrical power, which is measured with the comparison method [16–18]. This formula allows the comparison of axes values in cases where actual cylindrical powers are different. *C*_*c*_ is taken as the weighting factor, because it is considered more accurate than the cylindrical power of the tested instrument [14–16].

Prism 7 (GraphPad Software, Inc) was used for the statistical analyses. While estimating the sample size, we considered that, if the difference between the matched pairs was 0.25 D, data from a minimum of 54 eyes would be required to reject the null hypothesis with a probability (power) of 0.95. When testing this hypothesis, the type 1 error probability is 0.05. In the present study, we enrolled 70 eyes of 70 patients. Because the SE values that were obtained via autorefraction with cycloplegia (our gold standard) were correlated (*r* = 0.874, *P* < 0.001) and not significantly different between the right and left eyes (−0.06 ± 0.66 D, *P*=0.418), only the left eye of each patient was used for all analyses. Measurements were compared using a one-way analysis of variance test with Tukey's multiple comparisons. Correlations between measurements were evaluated with Pearson's correlation test (*r*). The correlations were defined as weak if *r* was below 0.3, moderate if *r* was between 0.3 and 0.7, and strong if *r* was higher than 0.7. Finally, agreement between the refraction measurement methods was investigated via Bland–Altman analysis. Statistical significance was defined as *P* < 0.05.

## 3. Results

**Figure 1:**
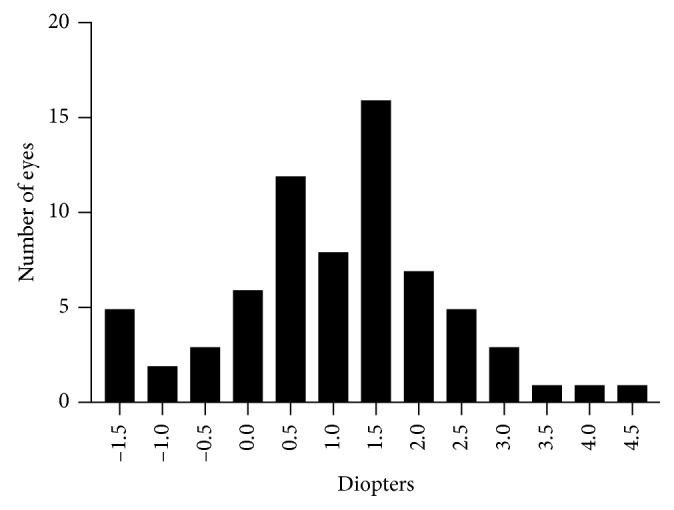
Frequency distribution of the spherical equivalent values of our study population.

Seventy children (29 girls and 41 boys) were recruited for this study. A total of 70 eyes were analysed. The mean age was 45.9 ± 11.4 months (range, 24–71 months).  Table 1 represents the mean spherical equivalent, mean spherical power, and mean cylindrical power values that were obtained by photorefraction without cycloplegia, photorefraction with cycloplegia, and autorefraction with cycloplegia. The frequency distribution of the spherical equivalent values of our study population is illustrated in Figure 1.

### 3.1. Comparison of Measurements between Photorefraction without Cycloplegia and Photorefraction with Cycloplegia

The spherical equivalent values that were acquired via photorefraction without cycloplegia and photorefraction with cycloplegia showed statistically significant differences (*P* < 0.001). The mean difference in spherical equivalents between the photorefraction without cycloplegia and photorefraction with cycloplegia was −1.17 ± 1.12 D (Plusoptix without cycloplegia showed myopic shift). The spherical power obtained via the photorefraction without cycloplegia and photorefraction with cycloplegia showed a statistically significant difference (*P* < 0.001). The difference in mean spherical power was −1.17 ± 1.15 D. The difference in cylindrical power that was obtained by the two methods was not statistically significant (*P* > 0.999), and the mean difference was 0.004 ± 0.36 D. We evaluated the accuracy of the axis when the cylindrical power was ≥0.25 D in measurements obtained from photorefraction without cycloplegia. In those cases, the mean axis difference was 0.30 ± 0.26 D. A weighted axis difference of 0.30 D is equal to 8.6 degrees of difference for 1 D. The spherical equivalent (*r* = 0.616; *P* < 0.001), spherical power (*r* = 0.533; *P* < 0.001), cylindrical power (*r* = 0.925; *P* < 0.001), and axis (*r* = 0.958; *P* < 0.001) measurements of photorefraction without cycloplegia were significantly correlated with photorefraction with cycloplegia. The 95% limits of agreement (LoA) between the two methods were as follows: spherical equivalent = −3.36 D to 1.03 D; spherical power = −3.43 D to 1.09 D; and cylindrical power = −0.71 D to 0.71 D.

### 3.2. Accuracy of Photorefraction without Cycloplegia

**Figure 2:**
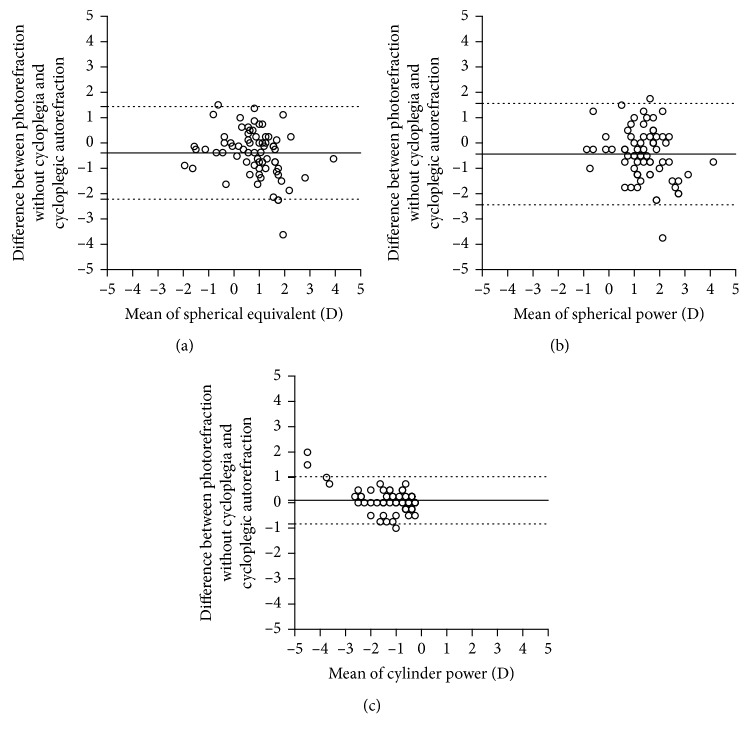
Bland–Altman plots illustrating the differences and means of values obtained with the photorefraction without cycloplegia and autorefraction with cycloplegia ((a) spherical equivalent, (b) spherical power, and (c) cylindrical power). The upper and the lower dashed lines represent 95% limits of agreement (calculated as the mean difference ± 1.96 × standard deviation); the solid line represents the mean difference between the methods (bias).

Table 2 shows the differences in mean spherical equivalent, mean spherical power, mean cylindrical power, and mean cylindrical axis values that were obtained by photorefraction without cycloplegia and autorefraction with cycloplegia. The spherical equivalent values that were acquired from photorefraction without cycloplegia and autorefraction with cycloplegia showed a statistically significant difference (*P*=0.021). The mean difference in the spherical equivalent between the photorefraction without cycloplegia and autorefraction with cycloplegia was −0.39 ± 0.93 D. The spherical powers obtained via photorefraction without cycloplegia and autorefractometer with cycloplegia showed a statistically significant difference (*P*=0.016). The difference in mean spherical power was −0.44 ± 1.02 D. There was no statistically significant difference in the cylindrical power obtained by the photorefraction without cycloplegia and autorefraction with cycloplegia (*P*=0.75). The mean cylindrical power difference was 0.10 ± 0.48 D. The accuracy of the axis was evaluated in cases where a cylinder power of ≥0.25 D had been determined using the autorefraction with cycloplegia. In those cases, the mean axis difference was 0.28 ± 0.30 D. A weighted axis difference of 0.28 D is equal to 8.1 degrees of difference for 1 D. Spherical equivalent (*r* = 0.688; *P* < 0.001), spherical power (*r* = 0.527; *P* < 0.001), cylindrical power (*r* = 0.917; *P* < 0.001), and axis (*r* = 0.838; *P* < 0.001) measurements of photorefraction without cycloplegia were correlated with autorefraction with cycloplegia. The LoA between the two methods were as follows: spherical equivalent = −2.22 D to 1.44 D; spherical power = −2.44 D to 1.56; and cylindrical power = −0.84 D to 1.03 D (Figure 2).

### 3.3. Accuracy of Photorefraction with Cycloplegia

**Figure 3:**
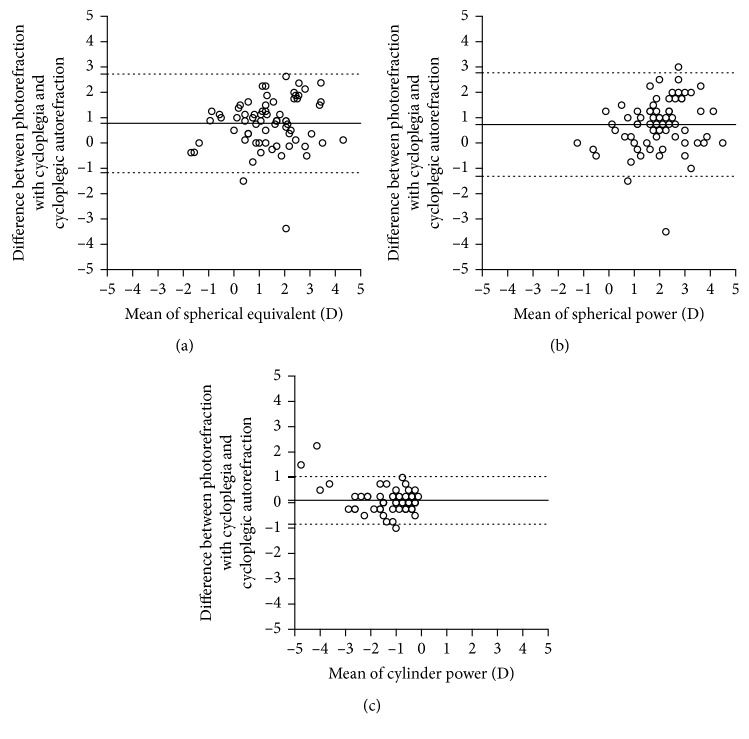
Bland–Altman plots illustrating the differences and means of values obtained with the photorefraction with cycloplegia and autorefraction with cycloplegia ((a) spherical equivalent, (b) spherical power, and (c) cylindrical power). The upper and the lower dashed lines represent 95% limits of agreement (calculated as the mean difference ± 1.96 × standard deviation); the solid line represents the mean difference between the methods (bias).

Table 3 shows the differences in mean spherical equivalent, mean spherical power, mean cylindrical power, and mean cylindrical axis values obtained via photorefraction with cycloplegia and autorefraction with cycloplegia. There was a statistically significant difference in spherical equivalent values obtained by the two methods (*P* < 0.001). The mean difference in spherical equivalent between the photorefraction with cycloplegia and autorefraction with cycloplegia was 0.78 ± 1.00 D. The difference in spherical power that was obtained by the photorefraction with cycloplegia and autorefraction with cycloplegia was statistically significant (*P* < 0.001). The difference in mean spherical power was 0.73 ± 1.04 D. The difference in cylindrical power that was obtained by the two methods was not statistically significant (*P*=0.79), and the mean difference was 0.09 ± 0.48 D. The accuracy of the axis was evaluated in the cases where a cylinder power of ≥0.25 D had been determined via autorefraction with cycloplegia. In those cases, the mean axis difference was 0.27 ± 0.25 D. A weighted axis difference of 0.27 D is equal to 7.8 degrees of difference for 1 D. Spherical equivalent (*r* = 0.728; *P* < 0.001), spherical power (*r* = 0.659; *P* < 0.001), cylindrical power (*r* = 0.908; *P* < 0.001), and axis (*r* = 0.852; *P* < 0.001) measurements of photorefraction with cycloplegia were correlated with autorefraction with cycloplegia. The LoA between the two methods were as follows: spherical equivalent = −1.17 D to 2.72 D; spherical power = −1.31 D to 2.77 D; and cylindrical power = −0.84 D to 1.03 D (Figure 3).

## 4. Discussion

For infant refraction, cycloplegic retinoscopy is the present gold standard. Nevertheless, in clinical and screening settings, photorefractors are being utilised with increasing frequency. Photorefractors are useful screening tools for working with infants and subjects with physical and/or mental disabilities [19]. They allow both eyes to be evaluated simultaneously and enable detection of refractive error at a distance that is less threatening to the infant. This allows for efficient testing and has been found to have excellent specificity and sensitivity [20, 21].

This study has shown that photorefraction using the Plusoptix A09 without cycloplegia causes a 0.39 D shift towards myopic values in preschool children due to accommodation when autorefraction with cycloplegia is applied as a gold standard comparison tool. Peyerols et al. [6] described a myopic shift of 0.52 D in a study population of 35 children with a mean age of 58 months. Racavy et al. [5] reported that photorefraction without cycloplegia in children between the ages of 7 and 12 years resulted in a greater myopic shift such as 1.21 D and 1.58 D in nonamblyogenic and amblyogenic hyperopia, respectively, which exceed the results found in our study. Because the increased myopic shift of photorefraction with increasing hyperopic values was also reported by Won et al. [7] (significant myopic shift only with a hyperopia of ≥3.00), the differences in mean SE between our study (0.98 ± 1.27 D) and that of Racavy et al.'s [5] study (the nonamblyogenic group = 3.62 ± 1.49 D; the amblyogenic group = 4.72 ± 1.2 D) as well as our study and that of Peyerols et al.'s [6] study (1.06 ± 2.04 D) might explain this disparity. We found that photorefraction without cycloplegia correlated moderately with cycloplegic autorefraction for spherical equivalent and spherical power. These results were similar to those of Won et al. [7], who had shown that spherical equivalent (*r* = 0.782; *P* < 0.001), spherical power (*r* = 0.748; *P* < 0.001), and cylindrical power (*r* = 0.893; *P* < 0.001) values that were obtained with photorefraction without cycloplegia strongly correlated with the measurements of the cycloplegic autorefractometer. The agreement between the SE values of these two methods was modest, with a wide 95% LoA (−2.22 to 1.44 D). Peyerols et al. [6] had also determined a wide 95% LoA (−1.55 D to 3.15 D) when they compared photorefraction without cycloplegia with cycloplegic autorefraction.

According to the results of our study, cycloplegia did not improve the accuracy of photorefraction. There was a statistically significant hyperopic shift in the spherical equivalent and spherical power values that were obtained by cycloplegic photorefraction. This result agrees with Yilmaz et al. [10] and Ozdemir et al. [11], who stated that cycloplegic photorefraction provides inaccurate results when assessing of spherical refractive errors in children. By contrast, Schimitzek and Lagrèze [9] suggested that photorefraction with cycloplegia measured spherical equivalence more accurately. The disparity between our results and the results of Schimitzek and Lagrèze [9] may have originated from a significant age difference in the study populations; their study population had a median age of 43 years, while our study population had a median age of 42 months. We found that photorefraction with cycloplegia correlated moderately with cycloplegic autorefraction for spherical equivalent and spherical power. The agreement between these two methods was modest, with wide 95% limits of agreement. The previous studies [9–11] evaluated neither 95% limits of agreement nor the correlation between these methods.

Our findings indicate that the cylindrical power and cylindrical axis values of photorefraction both with and without cycloplegia were in good agreement with autorefraction with cycloplegia. Similarly, recent studies have stated that values of cylindrical power and axes that were acquired from the photorefraction were moderately correlated with those acquired from cycloplegic retinoscopy [22] and cylindrical power that were acquired from cycloplegic and noncycloplegic autorefractometers [7].

Due to concerns regarding possible systemic side effects, the utilisation of cyclopentolate for routine eye examination in infants is avoided by some clinicians. Furthermore, tropicamide has been known historically to have an unsatisfactory cycloplegic performance in comparison with cyclopentolate. On the other hand, several recent studies have suggested that the cycloplegic effect provided by tropicamide has been adequate for the assessment of refractive error [12, 13]. Additionally, Yolton et al. [23] found no systemic side effects of tropicamide in an evaluation of 15,000 diagnostic pharmaceutical applications and reported that tropicamide was a safe agent. Applying eye drops inherently induces crying and agitation in most infants, but this agitation happens 25 minutes before the photorefraction procedure, not at the same time. Almost all infants would be calm enough by then to undergo refractive examination. For the above-listed reasons, 1% tropicamide has been used routinely in our eye clinic for refraction or dilated fundus examination in children.

The limitations of this study are as follows. The refractive error measurements that were derived from cycloplegic retinoscopy, which have been utilised as the gold standard to measure refractive error in most studies, were not present in this study. We used autorefraction with cycloplegia as the gold standard instead. This might be criticised, but the validity of autorefraction with cycloplegia for children refractive examination has been confirmed previously [3, 4]. In addition, a single measurement was performed on all patients because repeatability of photorefraction has been reported [5, 24]. Finally, because this study included only seven eyes with high hyperopia (≥3.00 D), generalisation of our results into this high amblyopia risk population might be limited.

Based on the results of our study, cycloplegia does not improve the accuracy of photorefraction in preschool children. For spherical power, spherical equivalent, cylindrical power, and cylindrical axis measurements, photorefraction without cycloplegia has moderate correlation with autorefraction with cycloplegia and seems to be a useful tool for screening purposes and for ophthalmologists who are not experienced with retinoscopy and in cases where autorefraction is not possible. On the other hand, since it shows myopic shift and has a wide LoA, photorefraction cannot replace the autorefraction, and the results should be interpreted prudently.

## Figures and Tables

**Table 1:** Mean spherical equivalent, spherical power, and cylindrical power values that were obtained by photorefraction without cycloplegia, photorefraction with cycloplegia, and autorefraction with cycloplegia.

	Spherical equivalent	Spherical power	Cylinder power
Photorefraction without cycloplegia (mean ± SD, D)	0.61 ± 1.03	1.16 ± 0.92	−1.11 ± 0.88
Photorefraction without cycloplegia (range, D)	−2.38 to 3.63	−1.25 to 3.75	−3.75 to −0.25
Photorefraction with cycloplegia (mean ± SD, D)	1.77 ± 1.40	2.33 ± 1.34	−1.11 ± 0.95
Photorefraction with cycloplegia (range, D)	−1.88 to 4.63	−1.25 to 4.75	−4.00 to 0
Autorefraction with cycloplegia (mean ± SD, D)	1.00 ± 1.27	1.60 ± 1.14	−1.21 ± 1.13
Autorefraction with cycloplegia (range, D)	−1.50 to 4.25	−1.25 to 4.50	−5.50 to 0

SD: standard deviation, D: diopters.

**Table 2:** Differences in mean spherical equivalent, mean spherical power, mean cylindrical power, and mean cylindrical axis (DA) values that were obtained by photorefraction without cycloplegia and autorefraction with cycloplegia.

Refractive parameter	Photorefraction without cycloplegia/autorefraction with cycloplegia		
	Mean ± SD	Range	*P*
DSE, D	−0.39 ± 0.93	–1.51 to 3.62	0.021^*∗*^
DS, D	−0.44 ± 1.02	–1.75 to 3.75	0.016^*∗*^
DC, D	0.10 ± 0.48	–2.00 to 1.00	0.75
DA, D	0.28 ± 0.30	0.00 to 2.18	NA

D: diopters; SD: standard deviation; DSE: difference in mean spherical equivalent; DS: difference in mean spherical power; DC: difference in mean cylindrical power; ^*∗*^*P* < 0.05; NA: not applicable.

**Table 3:** Differences in mean spherical equivalent, mean spherical power, mean cylindrical power, and mean cylindrical axis values obtained that were by photorefraction with cycloplegia and autorefraction with cycloplegia.

Refractive parameter	Photorefraction with cycloplegia/autorefraction with cycloplegia		
	Mean ± SD	Range	*P*
DSE, D	0.78 ± 1.00	–2.63 to 3.37	<0.001^*∗*^
DS, D	0.73 ± 1.04	–3.00 to 3.50	<0.001^*∗*^
DC, D	0.09 ± 0.48	–2.25 to 1.00	0.79
DA, D	0.27 ± 0.25	0.00 to 0.97	NA

D: diopters; SD: standard deviation; DSE: difference in mean spherical equivalent; DS: difference in mean spherical power; DC: difference in mean cylindrical power; ^*∗*^*P* < 0.05; NA: not applicable.

## Data Availability

The data used to support the findings of this study are available from the corresponding author upon request.

## References

[1] Repka M. X., Kraker R. T., Holmes J. M. (2014). Atropine vs patching for treatment of moderate amblyopia. *JAMA Ophthalmology*.

[2] Safir A. (1971). Retinoscopy. *International Ophthalmology Clinics*.

[3] Choong Y. F., Chen A. H., Goh P. P. (2006). A comparison of autorefraction and subjective refraction with and without cycloplegia in primary school children. *American Journal of Ophthalmology*.

[4] Prabakaran S., Dirani M., Chia A. (2009). Cycloplegic refraction in preschool children: comparisons between the hand-held autorefractor, table-mounted autorefractor and retinoscopy. *Ophthalmic and Physiological Optics*.

[5] Rajavi Z., Sabbaghi H., Baghini A. S., Yaseri M., Sheibani K., Norouzi G. (2015). Accuracy and repeatability of refractive error measurements by photorefractometry. *Journal of Ophthalmic and Vision Research*.

[6] Payerols A., Eliaou C., Trezeguet V., Villain M., Daien V. (2016). Accuracy of PlusOptix A09 distance refraction in pediatric myopia and hyperopia. *BMC Ophthalmology*.

[7] Won J. Y., Shin H. Y., Kim S. Y., Lee Y. C. (2016). A comparison of the Plusoptix S09 with an autorefractometer of noncycloplegics and cycloplegics in children. *Medicine*.

[8] Fogel-Levin M., Doron R., Wygnanski-Jaffe T., Ancri O., Ben Zion I. (2016). A comparison of plusoptiX A12 measurements with cycloplegic refraction. *Journal of American Association for Pediatric Ophthalmology and Strabismus*.

[9] Schimitzek T., Lagrèze W. A. (2005). Accuracy of a new photorefractometer in young and adult patients. *Graefe’s Archive for Clinical and Experimental Ophthalmology*.

[10] Yilmaz A. K., Uretmen O., Kose S. (2011). Accuracy of Plusoptix S04 in children and teens. *Canadian Journal of Ophthalmology*.

[11] Ozdemir O., Özen Tunay Z., Petriçli I. S., Ergintürk Acar D., Erol M. K. (2015). Comparison of non-cycloplegic photorefraction, cycloplegic photorefraction and cycloplegic retinoscopy in children. *International Journal of Ophthalmology*.

[12] Egashira S. M., Kish L. L., Twelker J. D., Mutt D. O., Zadnik K., Adams A. J. (1993). Comparison of cyclopentolate versus tropicamide cycloplegia in children. *Optometry and Vision Science*.

[13] Twelker J. D., Mutti D. O. (2001). Retinoscopy in infants using a near noncycloplegic technique, cycloplegia with tropicamide 1%, and cycloplegia with cyclopentolate 1%. *Optometry and Vision Science*.

[14] Erdurmus M., Yagci R., Karadag R., Durmus M. (2007). A comparison of photorefraction and retinoscopy in children. *Journal of American Association for Pediatric Ophthalmology and Strabismus*.

[15] Wesemann W., Rassow B. (1987). Automatic infrared refractors-A comparative study. *Optometry and Vision Science*.

[16] Schimitzek T., Wesemann W. (2002). Clinical evaluation of refraction using a handheld wavefront autorefractor in young and adult patients. *Journal of Cataract & Refractive Surgery*.

[17] Wesemann W., Rassow B. (1986). Modern instruments for subjective refraction. *Ophthalmology*.

[18] Wesemann W., Dick B. (2000). Accuracy and accommodation capability of a handheld autorefractor. *Journal of Cataract & Refractive Surgery*.

[19] Peterseim M. M. W., Papa C. E., Wilson M. E. (2014). Photoscreeners in the pediatric eye office: compared testability and refractions on high-risk children. *American Journal of Ophthalmology*.

[20] Matta N. S., Singman E. L., Silbert D. I. (2008). Performance of the Plusoptix vision screener for the detection of amblyopia risk factors in children. *Journal of American Association for Pediatric Ophthalmology and Strabismus*.

[21] Bloomberg J. D., Suh D. W. (2013). The accuracy of the plusoptiX A08 photoscreener in detecting risk factors for amblyopia in central Iowa. *Journal of American Association for Pediatric Ophthalmology and Strabismus*.

[22] Demirci G., Arslan B., Özsütçü M., Eliaçık M., Gulkilik G. (2014). Comparison of photorefraction, autorefractometry and retinoscopy in children. *International Ophthalmology*.

[23] Yolton D. P., Kandel J. S., Yolton R. L. (1980). Diagnostic pharmaceutical agents: side effects encountered in a study of 15,000 applications. *Journal of the American Optometric Association*.

[24] Yilmaz I., Ozkaya A., Alkin Z., Ozbengi S., Yazici A. T., Demirok A. (2015). Comparison of the plusoptix A09 and retinomax K-plus 3 with retinoscopy in children. *Journal of Pediatric Ophthalmology & Strabismus*.

